# Molecular Evolution and Inheritance Pattern of *Sox* Gene Family among Bovidae

**DOI:** 10.3390/genes13101783

**Published:** 2022-10-02

**Authors:** Mabel O. Akinyemi, Jessica Finucan, Anastasia Grytsay, Osamede H. Osaiyuwu, Muyiwa S. Adegbaju, Ibukun M. Ogunade, Bolaji N. Thomas, Sunday O. Peters, Olanrewaju B. Morenikeji

**Affiliations:** 1 Department of Biological Sciences, Fairleigh Dickinson University, Madison, NJ 07940, USA; 2 Division of Biological and Health Sciences, University of Pittsburgh, Bradford, PA 16701, USA; 3 Department of Animal Science, Faculty of Agriculture, University of Ibadan, Ibadan 200005, Nigeria; 4 Institute for Plant Biotechnology, Stellenbosch University, Stellenbosch 7600, South Africa; 5 Division of Animal and Nutritional Science, West Virginia University, Morgantown, WV 26505, USA; 6 Department of Biomedical Sciences, Rochester Institute of Technology, Rochester, NY 14623, USA; 7 Department of Animal Science, Berry College, Mount Berry, GA 30149, USA

**Keywords:** *Sox* gene, evolution, Bovidae, inheritance, transcription factors

## Abstract

*Sox* genes are an evolutionarily conserved family of transcription factors that play important roles in cellular differentiation and numerous complex developmental processes. In vertebrates, *Sox* proteins are required for cell fate decisions, morphogenesis, and the control of self-renewal in embryonic and adult stem cells. The *Sox* gene family has been well-studied in multiple species including humans but there has been scanty or no research into Bovidae. In this study, we conducted a detailed evolutionary analysis of this gene family in Bovidae, including their physicochemical properties, biological functions, and patterns of inheritance. We performed a genome-wide cataloguing procedure to explore the *Sox* gene family using multiple bioinformatics tools. Our analysis revealed a significant inheritance pattern including conserved motifs that are critical to the ability of *Sox* proteins to interact with the regulatory regions of target genes and orchestrate multiple developmental and physiological processes. Importantly, we report an important conserved motif, EFDQYL/ELDQYL, found in the *Sox*E and *Sox*F groups but not in other *Sox* groups. Further analysis revealed that this motif sequence accounts for the binding and transactivation potential of *Sox* proteins. The degree of protein–protein interaction showed significant interactions among *Sox* genes and related genes implicated in embryonic development and the regulation of cell differentiation. We conclude that the *Sox* gene family uniquely evolved in Bovidae, with a few exhibiting important motifs that drive several developmental and physiological processes.

## 1. Introduction

The *Sox* genes belong to a large group of genes whose DNA binding domain is called a high mobility group (HMG), encoding diverse and well-conserved transcription factors, and consisting of 20 genes in vertebrates and only a handful in invertebrates [1,2]. *Sox* genes share homology with the HMG box of the sex-determining region Y (*Sry*) gene, a founding member of the *Sox* gene family, which is required to specify the male phenotype. This group of genes owes their acronym to sharing 46% or more identity to the *Sry* gene in the HMG box [3].

Vertebrate *Sox* genes have been divided into eight groups (A–H) based on conserved protein domains and similarity in their amino acid sequences [2,4]. Members of the same group are highly similar to each other within and outside the HMG box, while those of different groups share a lower degree of identity in the HMG box and no significant identity outside the domain [3]. Most members of this group are scattered throughout the genome, with the exception of *Sry* and *Sox*3, which are located on sex chromosomes [2].

*Sox* proteins control transcription in several ways; for example, the protein domain connects the minor groove of DNA to AACAAAG, AACAAT, and related sequences and induces a sharp bend in DNA, thereby allowing the protein to play an important structural role in the assembly of transcription enhancer complexes [3]. They exhibit their structural role by shaping the regulatory regions and establishing physical contacts between transcription factors bound to the same target gene promoter or enhancer [2]. Thus, the regulatory functions of *Sox* proteins require the cooperation of interacting partners that bind DNA and enable the specific selection of target genes. This type of cooperation is dynamic, allowing *Sox* proteins to regulate different events by changing partner factors—a key factor that drives the progression of developmental processes [5].

All *Sox* genes have a specific expression pattern and mostly play important roles in the determination of cells fate and the differentiation of cells into specific lineages, such as embryonic stem cells, neuronal and glial cells, Sertoli cells, chondrocytes, etc. [3]. They have also been shown to be capable of reprogramming differentiated somatic cells into pluripotent stem cells [6,7], activating genes that are important for maintaining the pluripotent cell state [8], and inducing the delta crystallin gene in the lens [9].

The functions of the members of the *Sox* B1 (*Sox*) family overlap, although each has a function during the migration of neuronal precursors in the ganglionic eminence [10]. *Sox*2 and *Sox*3 deficiency cause pituitary defects [11]. Single knockouts of *Sox*5 or *Sox*6 in mice lead to some skeletal abnormalities, and a *Sox*5 and *Sox*6 double knockout leads to a lack of chondrogenesis [12]. Mutations in *Sox*18 induce severe cardiovascular and hair follicle defects including lymphatic dysfunction in developing embryos [13,14]. Whereas mutational disruptions in *Sox*9 lead to the failure of sertotic placode invagination [15]. Members of the *Sox*F subfamily exhibit overlapping functions [16] and are expressed in endothelial cells, while their mutation has been reported in lymphatic defects [17]. Kamai-Azuma et al. [18] also reported defects in gut tube formation in *Sox*17 knockout mice. Members of the *Sox*C subfamily (*Sox*4, 11, and 12) are expressed in neural and mesenchymal progenitor cells. Mutations in these subfamilies lead to the malformation of various organs. *Sox*4 and *Sox*11 knockout mice exhibit multiple organ defects [19]. Several studies aimed at discovering more *Sox* protein functions have been impaired by extensive functional redundancy among members of the same group and pleiotropy [14].

The Bovidae family includes several economically and socially important ruminant species such as cattle, sheep, goats, and antelope, comprising more than 140 recognized species distributed among 49 genera. The evolution of the different members of this family has been molded by several mechanisms including temperature adaptations, feeding ecology, vegetation physiognomy, climate fluctuations, immigration, adaptive radiations, and mass extinctions [20]. There are published reports examining the evolutionary history of Bovidae through morphological data, allozymes, serum immunology, DNA sequences, and mitochondrial DNA analyses [21]. Protein and DNA-based sequence methods utilize sequence variations and the analysis of conserved domains, in addition to computational methods utilizing genetic variation within and between amino acid sequences to predict functional and structural outcomes [22,23,24,25].

Most computational analyses on *Sox* genes have focused on individual genes and their regulatory functions rather than the entire gene family. Despite the abundance of information on the importance of *Sox* proteins in various developmental processes in other species, we found no published report on the *Sox* gene family in Bovidae. Taking this into consideration along with the fact that *Sox* genes are significant players in the regulation of developmental processes, we examined the evolutionary dynamics of the *Sox* gene family within Bovidae by evaluating their biochemical properties, structural prediction, conserved domains, protein–protein interaction, and phylogenetic relationships.

## 2. Materials and Methods

### 2.1. Sequence Retrieval and Multiple Sequence Alignment

*Sox* gene sequences of the Bovidae family, namely, *Bos taurus* (Cattle), *Bos grunniens* (Domestic Yak), *Bison bison* (Bison), *Capra hircus* (Goat), *Ovis aries* (Sheep), *Bubalus bubalis* (Buffalo), *Bos mutus* (Wild Yak), and *Antilocapra americana* (Antelope) species, were employed for comparative analysis. All sequences were retrieved in the FASTA format from the National Centre for Biotechnology Information (NCBI) database (https://www.ncbi.nlm.nih.gov accessed on 10 April 2022). The *Bos* sp. accession numbers are as follows: *Sox*A (ABY19364.1), *Sox*B (XP_005214070.1, NP_001098933.1, XP_027390716.1), *Sox*B2(NP_001157253.1,XP_024855919.1}, *Sox*C (NP_001071596.1,XP_024855327.1,XP_010809935.2), *Sox*D (AAI14659.1,XP_027419183.1, XP_027419995.1) *Sox*E (XP_002698019.3,XP_027374751.1, XP_027396627.1), *Sox*F (XP_027404803.1, XP_027416795.1, XP_027415027.1), *Sox*G (XP_027374313.1), and *Sox*H (XP_027401619). The total number of *Sox* genes, categories, and abundance of each subfamily were collated for further analysis.

Sequence alignment was performed using the ClustalW algorithm on the MEGAX software [26]. The alignment was imported to itol for phylogenetic tree construction using Maximum Likelihood Tree method, as described in [27]. Thereafter, MEME (Multiple Em for Motif Elicitation http://alternate.meme-suite.org accessed on 12 June 2022) version 5.0.2 [28] was used to predict the conserved motif structures encoded across all *Bos Sox* genes. All sequences were scanned for the presence of the sequence motif RPMNAFMVW, which has been reported to be conserved for all *Sox* sequences other than Sry and only sequences that have the motif were included in further analysis. We used Multalin software (http://multalin.toulouse.inra.fr/multalin accessed on 24 April 2022) to identify the conserved amino acid sequences among the species. The sequence logo of the identified domain in the *Sox* protein family was constructed with WebLogo (http://weblogo.berkeley.edu/logo.cgi, accessed on 24 April 2022).

### 2.2. Physicochemical Characterization of Sox Gene Family

To decipher the physicochemical properties of *Sox* gene family in Bovidae, we used Expasy ProtParam server (https://web.expasy.org/protparam accessed on 21 June 2022) to compute the molecular weight, isoelectric point (pI), instability index, aliphatic index, and grand average of hydropathicity (GRAVY) of all the proteins in the Bovidae *Sox* family, as previously described [23]. The chromosomal location of each *Sox* gene was retrieved from Unitprot/KB/Swiss-Prot database in NCBI (https://www.ncbi.nlm.nih.gov accessed on 10 April 2022).

### 2.3. Identification of Interacting Proteins, Functional Enrichment, and Pathway Analysis

Protein–protein interactions were predicted using the Search Tool for the Retrieval of Interacting Genes database (STRING https://stringdb.org accessed on 7 June 2022). This is important to elucidate the association of *Sox* proteins with other molecules. Functional enrichment analysis was performed with PANTHER (http://www.pantherdb.org accessed on 5 June 2022) [29] and Database for Annotation, Visualization, and Integrated Discovery (DAVID) to classify the genes according to their function, annotated with ontology terms: biological processes, cellular components, and molecular functions of the studied genes.

### 2.4. Evolutionary Phylogenetic Analysis of Sox Gene Family

To study the evolutionary pattern and inheritance of *Sox* genes in *Bos* sp., a multiple sequence alignment was performed using ClustalW tool in MEGA11 [26] with default parameters; all positions containing gaps and missing data were eliminated. The phylogenetic trees of *Sox* gene family in *Bos* sp. were constructed by adopting the Neighbor-joining method of MEGA11 software with the following parameters: Poisson correction, pairwise deletion, and 1000 bootstrap replicates. The constructed tree files were visualized using *itol* (Interactive Tree of Life, https://itol.embl.de accessed on 3 June 2022).

## 3. Results

### 3.1. Genome Wide Analysis of Sox Genes in Bovidae

**Figure 1:**
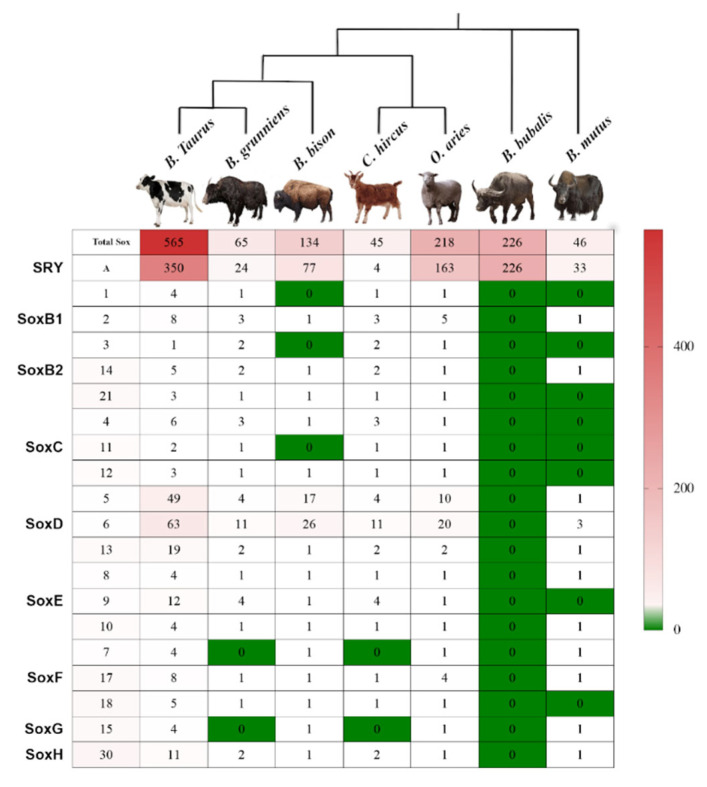
The phylogenetic analysis, categories, and abundance of the *Sox* gene family in Bovidae.

A total of 350 variants of *SRY*, 49 variants of *Sox*5, and 63 variants of *Sox* 6 were reported in *B. taurus* (Figure 1). *Bos mutus* has 33 variants of *Sox*A and 3 variants of *Sox* 6. The water buffalo (*B. bubalis*) has 226 variants of *Sox*A and 34 variants of *Sox*6, while 24 variants of *Sox*A were reported for Yaks. Sheep (*O. aries*) have 163 variants of *Sox*A and 20 variants of *Sox*6 while goats (*C. hircus*) presented with 4 variants of *Sox*A and 11 variants of *Sox*6. *B. bison* has 77 variants of *Sox*A and 26 variants of *Sox*6. There was no record of *Sox* variants for antelopes.

### 3.2. Physicochemical Properties of Sox Genes

Our analysis of the physicochemical properties revealed that *Sox*15 had the lowest molecular weight of 25,140 Da, while the *Sox*D family [5,6,13] had the highest molecular weight values (Table 1). The *pI* is the pH at which a particular molecule carries no net electric charge; this value is useful for understanding protein charge stability. Overall, the pI ranged from 4.95 to 9.85 (Table 1). The results for the instability index reveal that the genes were all unstable (II > 40), with the lowest value of 41.96 in *Sox*1 and the highest of 80.78 in *Sox*9. The aliphatic index, regarded as the factor determining the thermostability of globular proteins, was lowest in *Sox*1 (44.44) and highest in *Sox*13 (71.88). A high extinction coefficient and low negative GRAVY values were also observed.

### 3.3. Evolutionary Dynamics and Phylogenetic Analysis

**Figure 2:**
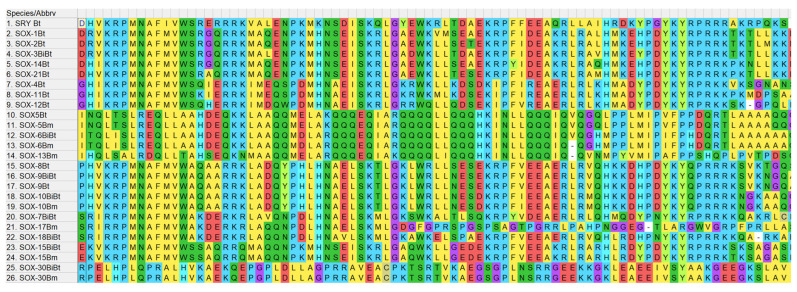
Multiple sequence alignment of *Sox* genes in *Bos* sp. (SRY: Sex-determining region Y, Bt: *Bos taurus*, BiBt: *Bos* indicus x *Bos tautus*, and Bm: *Bos mutus*. Colors were assigned by MEGA software based on the biochemical properties of the amino acids. Red, acidic; yellow, non-polar neutral; cyan, positive polar basic; blue, positive polar; green, neutral polar; purple, neutral non-polar; lime, neutral polar).

Furthermore, this study examined the evolutionary pattern of 31 *Sox* sequences of the *Bos* sp. The multiple sequence alignment indicated that Lysine (K), Proline (P), Arginine R), Glycine (G), and Leucine (L) are conserved in the *Sox* family (Figure 2). The sequences demonstrated significant variability in both percentage identity and similarity in *Bos* despite their common evolutionary origin. The analysis of the percentage identity and similarity revealed that *Sox*14 and 21 (71.73%) were highly similar while *Sox*7 and 30 exhibited the lowest similarity (15.17%) (Table 2 and Supplementary Table S1)

**Figure 3:**
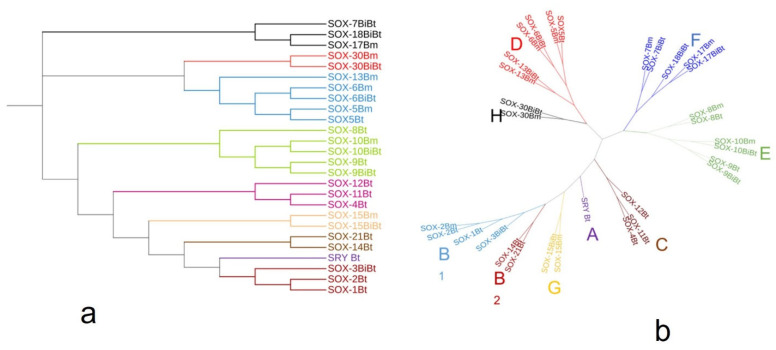
(**a**) Rooted phylogeny of *Sox* genes in *Bos* sp. computed using the neighbor-joining method (MEGA). (**b**) Unrooted phylogeny of *Sox* genes created by the interactive tree of life (ITOL) online software (https://itol.embl.de accessed on 3 June 2022) which assigned color to the different subfamilies. The various subfamilies (A, B1, B2, C, D, E, F, G and H) are highlighted in different colors.

**Figure 4:**
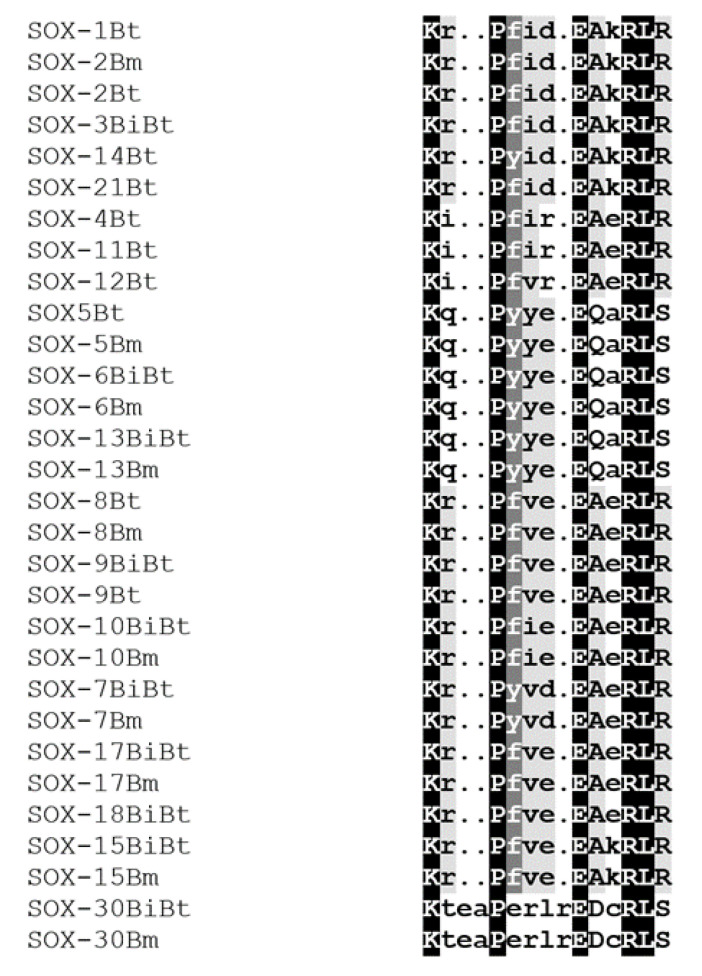
Conserved domain pattern in *Sox* proteins across *Bos* sp.

**Figure 5:**
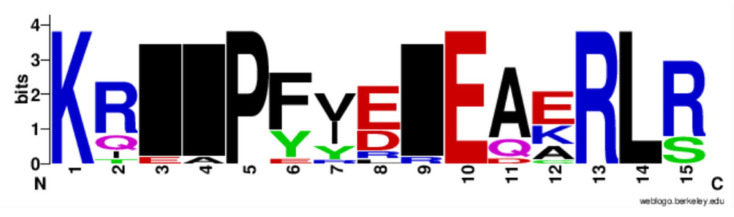
Logo plots of *Sox* protein sequences displaying the most conserved domain and positions of amino acids. A total of 26 multiple sequence alignments are represented using Weblogo (http://weblogo.berkeley.edu/logo.cgir accessed on 24 April 2022). The relative frequency of the amino acids is shown on the *y*-axis.

Using the distance-based method, we examined the phylogenetic relationships among the *Bos* *Sox* sequences. A phylogenetic tree based on the alignment showed that the *Sox* proteins are segregated into nine specific groups: A (*SRY*), B_1_ (*Sox*1, 2, and 3), B_2_ (*Sox*14 and 21), C (*Sox*4, 11, and 12), D (*Sox*5, 6, and 13), E (*Sox*8, 9, and 10), F (*Sox*7, 17, and 18), and *Sox*15 and 30 are the sole members of Groups G and H, respectively (Figure 3a). The branch lengths, representative of the extent of divergence, suggest a monophyletic arrangement, with *Sox*B_1_ diverging first. The result is further represented in an unrooted tree, with the expected clustering pattern observed for all groups (Figure 3b). Figure 4 shows the MSA of the homology of the HMG domain across the *Sox* genes showing that Lysine (K), Proline (P), Glutamate (E), Arginine (R), and Leucine (L) are 100% conserved in this region. The sequence logo showing the relative frequencies of each of the conserved domains and their respective positions is displayed (Figure 5).

### 3.4. Conserved Motif Analysis of Sox Genes

**Figure 6:**
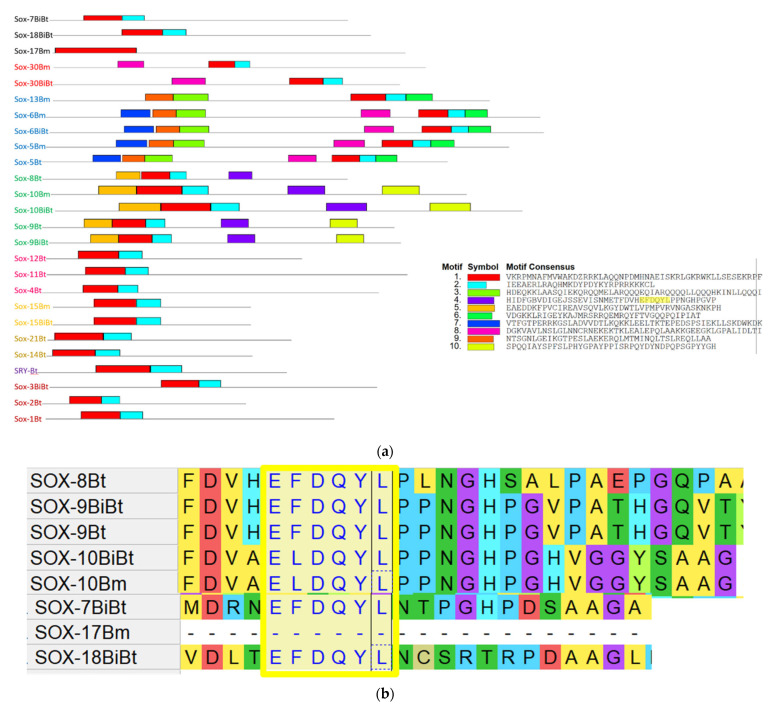
(**a**) Motif patterns of *Bos* *Sox* gene family. Ten differentially conserved motifs are indicated with differently colored boxes using the online Multiple Expectation Maximization for Motifs Elicitation (MEME, http://alternate.meme-suite.org accessed on 12 June 2022) program. M4 included an important motif, which was highlighted in yellow. (**b**) Multiple sequence alignment of *Sox* genes in *Bos* sp. (Bt: *Bos taurus*, BiBt: *Bos* indicus x *Bos tautus*, and Bm: *Bos mutus*) showing conserved transactivation motif highlighted in yellow. The motif is conserved in *Sox* 7–10 and 18 but not *Sox* 17. Amino acids colors were assigned by MEGA software based on their biochemical properties. Red, acidic; yellow, non-polar neutral; cyan, positive polar basic; blue, positive polar; green, neutral polar; purple, neutral non-polar; lime, neutral polar).

To further characterize the evolutionary pattern of the *Sox* gene in the *Bos* species, a conserved motif analysis was performed using the MEME and MEGA programs. The results indicate that 10 conserved motifs are present in the *Bos* species. Interestingly, we found that conserved motifs 1 and 2 (M1 and M2) exist in all members of the *Sox* family except *Sox*17, which had motif 1 only. The other nine motifs were present in the different *Sox* groups. M1 and M2 are the core HMG boxes, containing 79 amino acid residues. As shown, five motifs are present in *Sox*D—*sox*13 and *Sox*E:*Sox*9 and 10, and seven motifs in *Sox*D:*Sox*5 (Figure 6a). Four motifs exist in *Sox*E:*Sox*8 and three motifs in *Sox*H:*Sox*30. Interestingly, we found an importantly conserved motif, namely, EFDQYL/ELDQYL, in both *Sox*E and F including *Sox* 7–10 and 18 with the exception of *Sox* 17 (Figure 6b). This motif is crucial for the transactivation and regulation of these protein family and has also been observed in humans.

### 3.5. Characterization of Functional Motifs

**Figure 7:**
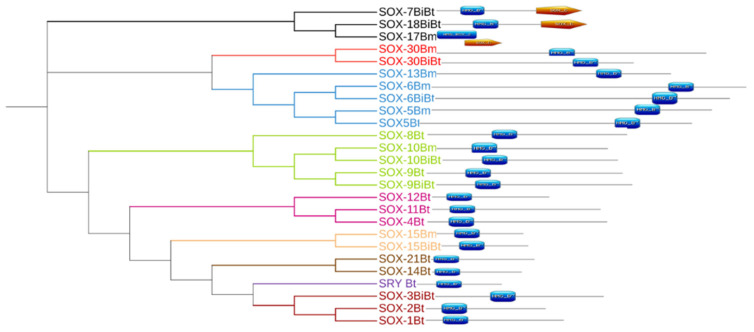
Comparison of intra-domain features of *Sox* proteins. This comparison shows the HMG box and *Sox*C domain that provide additional information about the structure and functions of Sox proteins. Members of *Sox*F subfamily have an additional C- terminal domain.

All the *Sox* sequences analyzed were individually scanned for matches against the PROSITE collection of protein signature databases. We found two main domains, HMG box B and the *Sox*C terminal domains (Figure 7), with varying frequency across the *Sox* gene family in Bovidae. We found one proline-rich domain in *Sox*9, 17, 18, 15, and 30. The amino acid composition analysis reveals that these proteins have the highest proline concentration among the studied *Sox* proteins (*Sox*9-16.98%; *Sox*17-16.34%; *Sox*18-19.28%; *Sox*15-14.59%; *Sox*30-14.40%) (Supplementary Table S2).

### 3.6. Biological, Molecular, and Cellular Function of Bos Sox Genes

**Figure 8:**
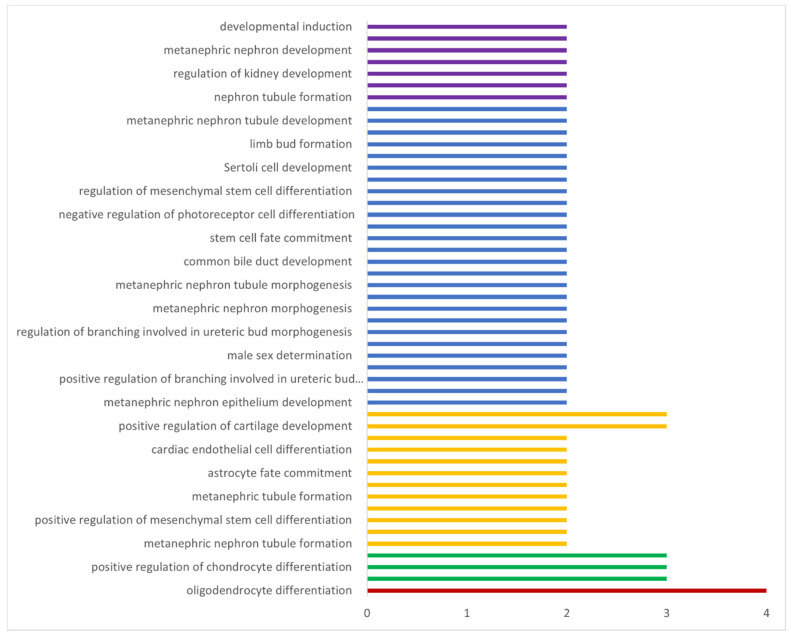
The biological process and the corresponding number of target genes involved. Red bars indicate the biological process/pathways predicted with the highest *p*-value; purple bars indicate the pathways predicted with the lowest *p*-value. Figure created with Microsoft Excel (2017) (https://www.microsoft.com/en-us/microsoft-365/excel accessed on 1 September 2022).

For the Gene Ontology analysis, three criteria were utilized: the biological processes the genes were involved in, the cellular components they are a part of, and their molecular function. We identified 49 biological processes with a false discovery rate <100, 6 cellular components, and 19 molecular functions (Table S3, Figure 8). The DAVID analysis revealed 26 biological processes, 2 cellular components, and 7 molecular functions (Supplementary Table S4). Nineteen significant biological processes including stem cell fate specification, the positive regulation of mesenchymal cells, renal vesicle induction, retinal rod cell differentiation, metanephric nephron tubule formation, the negative regulation of myoblast differentiation, and the positive regulation of chondrocyte differentiation were concomitantly identified from both databases (Supplentary Tables S3 and S4, Figure 8).

### 3.7. Protein–Protein Interaction Cluster with Sox Genes

**Figure 9:**
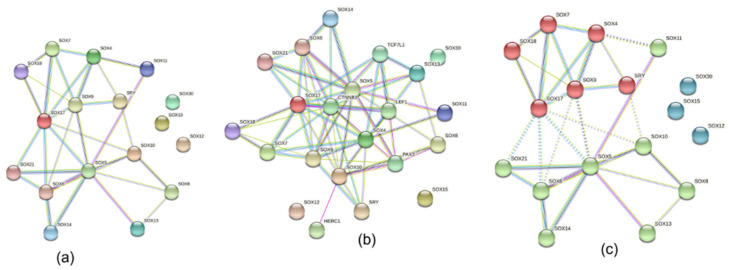
*Sox* protein interaction. Protein–protein interaction: (**a**) *Sox* proteins only; (**b**) predicted interactions with other proteins; (**c**) k means clustering. Network created using STRING (string-db.org).

In order to analyze the protein–protein interaction (PPI), co-expression, co-regulation, and physical association, we used STRING to build the protein network. Using k-means clustering we generated three clusters composed of closely connected interactions. The results revealed a major PPI network cluster with all the studied genes except *Sox*12, 15, and 30, which exhibited no interaction (Figure 9a). *Sox*5 had the most interaction with 10 nodes while *Sox*11, 13, and 14 had the least interaction. However, *Sox*1, 2, and 3 were not found in the cluster.

The PPI also indicated a possible involvement in pathways such as metabolic, developmental, and regulatory. Cellular signaling pathways such as the regulation of the Wnt signaling pathway, the negative regulation of the canonical Wnt signaling pathway, the regulation of the canonical Wnt signaling pathway, and the signal transduction involved in cell cycle checkpoint were associated with these protein networks. The STRING-extended analysis revealed the interaction of specific *Sox* genes with CTNNB1, HERC1, LEF1, PAX3, and TCF7L1 (Figure 9b).

## 4. Discussion

The *Sox* family of transcription factors exhibit diverse tissue-specific expression patterns during early embryonic development and plays important roles in cell fate [30]. To provide a deeper understanding of the inheritance pattern and the role of *Sox* protein in Bovidae evolution, we utilized sequences available in public databases to perform several computational analyses, yielding evolutionary relationships among the *Sox* gene family in *Bos*. Our analysis identified nine distinct clades, which were assigned to the known *Sox* groups A–H [31,32]. The genes within the same subgroup shared greater sequence similarity outside the HMG domain than those that are more related, a finding consistent with a previous report [33]. We observed high percentages of proline, glycine, and alanine in all the *Sox* proteins. Studies have shown that proline readily adopts a cis and trans configuration in response to subtle influences, induces sharp turns in the local geometry, is important in protein–protein interaction and cell adhesion, and mediates signal transduction [34]. The *Sox*G and *Sox*D subfamilies had the lowest and highest amino acid residues and molecular weights, respectively. All the *Sox* proteins had negative GRAVY values, indicating the hydrophilic nature of the proteins, as previously described [31,35], enhancing their binding capacity [36,37]

*Sox*A (*SRY*) protein, a transcriptional regulator that controls the genetic switch in male development, is the only member of Group A and formed a monophyletic group in the phylogenetic analysis. Its expression inhibition causes defects in the development of testes [32,38], with cytogenetic and molecular studies revealing that it possibly arose from a mutation of *Sox*3 and the fusion of the gene with regulatory sequences from another gene already on the X chromosome [39]. STRING pathway analysis revealed its association with *Sox*9, with GO enrichment confirming that SRY and *Sox*9 are enriched in male sex determination, which is consistent with published reports [31,40]. *SRY* binds to the TESCO (testis-specific enhancer core) sequence of *Sox*9 and initiates the differentiation of somatic precursors into Sertoli cells that coordinate the gonadal development into the testis [41]. Despite the substantial variation in expression profile structure and amino acid sequences within mammals, the function of *SRY* to activate *Sox*9 during development appears to be conserved.

The *Sox* B_1_ group comprises *Sox*1, 2, and 3, which are groups of transcription factors that have been reported to affect the Wnt β-catenin signaling pathway [2]. *Sox*1 inhibits β-catenin/TCF transcription activity by binding to β-catenin via their C-terminal region while *Sox*2 overexpression down-regulates the Wnt β-catenin signaling pathway suggesting a negative feedback loop between them. These proteins also promote the self-renewal of neural progenitor cells. PANTHER analysis shows that *Sox*3 interacts with *SRY* and *Sox*9 in sex determination. There was no interaction reported for *Sox*1 and 2, possibly attributed to a functional redundancy in this subfamily [42,43]

The proteins encoded by members of *Sox*B_2_ subfamily, *Sox*14 and *Sox*21, are very similar to each other but different from subfamily B_1_ in areas outside the HMG domain. They have been implicated in the regulation of mesenchymal stem cell differentiation. *Sox*21 has been reported to promote neurogenesis in mice while *Sox*14 mediates terminal differentiation of the neural progenitor cells [44]. The STRING pathway analysis identified *Sox*14 as one of the genes involved in the regulation of neurogenesis while *Sox*21 was enriched in stem cell differentiation.

The *Sox*C subfamily (*Sox*4, 11, and 12) has three members in most vertebrates [3]. *Sox*4 and 11 share a similar function and structural properties. Reports on *Sox*12 functional properties are scarce. *Sox*4 and 11 were shown to be involved in lymphocyte differentiation, osteoblast development, neural and glial cell development, control progenitor development, and the capacity to crosstalk with other cells to grow and mature skeletal structures [1,3]. Phylogenetic analysis revealed that the three proteins are closely related, as they all appeared in one clade. PROSITE revealed a *Sox*C-rich domain in all *Sox*F genes, suggesting that the complex interacts with this group. The HMG box domain in this group is virtually identical; therefore, the difference in DNA binding efficiency may be due to sequence differences outside the HMG box [3]. The STRING pathway analysis identified *Sox*4 and *Sox*11 as enriched in cardiac ventricle formation and limb bud formation, enteric nervous system development, and glial cell proliferation.

The *Sox*D subfamily (*Sox*5, 6, and 13) have higher amounts of amino acid residues than other *Sox* proteins. Our result showed that this subgroup is significantly enriched in oligodendrocyte differentiation, cell fate commitment, and glial cell differentiation. Members of this group formed a paraphyletic group with *Sox*30 suggesting a recent divergence. These genes interact with *Sox*9 of the *Sox*E subfamily, and expression of the three *Sox* genes culminates in growth plate proliferation in chondrocytes. Furthermore, the co-inactivation of *Sox*5 and 6 genes was reported to result in stunted or underdeveloped growth plates and articular cartilage, while *Sox*9 is required to turn on and maintain chondrocyte-specific genes [45,46].

*Sox*E proteins, *Sox*8, 9, and 10, overlap in expression, play critical roles in various biological processes, and are renowned for their role in transactivation. Haseeb and Lefebvre [47] reported a study in humans wherein *Sox*E and F proteins possess the unique motif EFDQYL/ELDQYL required for transactivation, which we also found in both *Sox*E and F of the Bovidae family. Based on its amino acid composition, it is functionally crucial and highly conserved in various vertebrate and invertebrate species [48]. The residues within the motif revealed the presence of acidic and hydrophilic amino acids (E, Q) alternating with hydrophobic residues (F, Y, L), which fold into pocket-like structures involved in recognizing and binding to functional partners and coactivators [47]. Other studies also showed that the sequence is required for the interaction of *Sox*9 and β-catenin, [49]

The *Sox*F subfamily promotes the fate specification of endothelial cells [50]. They are thought to share a recent common ancestor with *Sox*E and exhibit a substantial overlap in expression function and regulation implying a redundant type of cooperation. The only *Sox* member in the G subfamily is *Sox*15. This *Sox* protein is found only in mammals and shares a common ancestry with *Sox*B, forming a paraphyletic tree. [14,51,52]. This common ancestry likely accounts for the ability of *Sox*15 to replace the function of *Sox*2 in the self-renewal of mouse stem cells [53,54]

The STRING analysis revealed the complex interaction of the studied *Sox* proteins with CTNNB1, PAX3, HERC1, and TCF7L1. The catenin β-1 gene (CTNNB1) provides instructions for making the β-catenin protein, which is primarily found at junctions connecting neighboring cells. β-catenin plays a major role in sticking cells (adhesion), cell signaling, and in cell communication [55]. This pathway is important for *Sox* proteins to recognize target genes during cellular interactions. CTNNB1 formed complex interactions with *Sox*2,6,7 and 9 in several developmental processes in *Coturnix japonica* [31]. Furthermore, CTNNB1 also interacts with *Sox*17 and 30, LEF1, and TCF7L1 in β-catenin binding. LEF1 (lymphoid enhancer-binding factor 1) shares significant homology with HMG protein and is involved in the Wnt canonical pathway, cell differentiation, and follicle morphogenesis [56]. Pax3, *Sox*10, and c-Ret are components of a neural crest development pathway, and the interruption of this pathway at various stages results in neural crest-related human genetic syndromes [57].

## 5. Conclusions

Our study shows the detailed molecular evolution, inheritance pattern, and functions of *Sox* genes in the *Bos* family using several computational tools. In addition, this study documents the first comprehensive evidence of genetic variation in the *Sox* gene family in *Bos* sp. We reported a total of 20 *Sox* genes, which were classified into nine subfamilies based on phylogenetic analysis. We also provided evidence of divergent evolution in some *Sox* genes among the *Bos* family. This study further revealed important functional motifs that drive these proteins and enhance their ability to shape the regulatory regions of several genes and promote cells’ fate and differentiation. Lastly, we found the motif EFDQYL/ELDQYL, which is required for the unique transactivation ability of *Sox*E and *Sox*F proteins. Based on the findings of this study, we have provided a suitable background for further studies to harness the potential of these protein families in immunotherapeutic and regenerative medicine targeted at Bovidae.

## Figures and Tables

**Table 1:** Physicochemical properties of *Sox* genes in *Bos* sp.

	Gene Symbol		Size	MW (Da)	pI	Chromosome	AI	II	GRAVY	Extinction Coefficient
		Accession Number								
*Sox*A	SRY	ABY19364.1	229	26,627.11	9.53	Y	62.62	60.71	−0.858	30,285
*Sox*B_1_	*Sox*1	XP_005214070.1	369	*	*	1	44.44	41.96	−0.472	35,870
	*Sox*2	NP_001098933.1	320	34,480.95	9.74	1	48.25	59.43	−0.739	37,360
	*Sox*3	XP_027390716.1	451	45,113.00	9.78	X	61.18	69.35	−0.314	38,850
*Sox*B_2_	*Sox*14	NP_001157253.1	240	26,485.39	9.68	1	63.17	53.51	−0.585	33,015
	*Sox*21	XP_024855919.1	277	28,666.92	9.74	12	69.13	58.51	−0.208	31,525
*Sox*C	*Sox*4	NP_001071596.1	481	47,785.11	7.20	23	54.22	58.41	−0.478	35,660
	*Sox*11	XP_024855327.1	451	47,147.03	4.95	11	58.16	65.27	−0.652	37,150
	*Sox*12	XP_010809935.2	314	33,997.76	5.14	13	49.87	67.74	−0.982	40,575
*Sox*D	*Sox*5	AAI14659.1	728	80,127.28	6.13	5	69.48	63.71	−0.746	43,445
	*Sox*6	XP_027419183.1	858	95,541.56	7.93	15	64.52	61.11	−0.794	64,790
	*Sox*13	XP_027419995.1	623	69,061.04	6.14	16	71.88	69.67	−0.742	41,870
*Sox*E	*Sox*8	XP_002698019.3	534	56,239.99	7.77	25	52.25	59.49	−0.708	63,175
	*Sox*9	XP_027374751.1	524	57,173.49	6.31	19	46.98	80.78	−0.984	55,935
	*Sox*10	XP_027396627.1	469	50,020.20	6.19	5	53.30	58.52	−0.822	54,445
*Sox*F	*Sox*7	XP_027404803.1	534	57,216.30	9.62	8	55.66	63.44	−0.834	58,830
	*Sox*17	XP_027416795.1	410	43,060.76	5.91	14	60.63	65.47	−0.509	39,225
	*Sox*18	XP_027415027.1	389	41,337.00	8.42	13	62.96	78.07	−0.573	40,255
*Sox*G	*Sox*15	XP_027374313.1	233	25,140.18	9.85	19	49.06	69.49	−0.875	38,515
*Sox*H	*Sox*30	XP_027401619.1	757	82,746.76	8.62	7	70.01	68.76	−0.603	48,415

* *Sox* 1 Sequence contains several consecutive undefined AA. Its pI and Mw cannot be computed.

**Table 2:** Molecular similarity index of *Sox* gene family in Bovidae.

SRY																			
***SOX*** **-1Bt**	50	100																	
***SOX*** **-2Bt**	51.80	74.05	100																
***SOX*** **-3BiBt**	51.80	68.62	68.20	100															
***SOX*** **-14Bt**	47.59	46.42	43.75	47.76	100														
***SOX*** **-21Bt**	42.77	45.88	44.35	43.92	71.42	100													
***SOX*** **-4Bt**	36.14	33.20	34.30	32.54	34.37	37.25	100												
***SOX*** **-11Bt**	36.74	31.12	33.05	32.54	34.82	32.15	54.47	100											
***SOX*** **-12Bt**	30.72	38.46	38.46	41.75	34.61	38.46	59.89	56.04	100										
***SOX*** **5Bt**	16.86	14.12	12.13	13.33	12.05	12.54	12.87	13.22	16.48	100									
***SOX*** **-5Bm**	16.86	14.12	12.13	13.33	12.05	12.54	12.83	13.22	16.48	100	100								
***SOX*** **-6BiBt**	19.27	14.88	14.22	13.72	15.62	11.37	12.35	14.78	15.38	81.43	81.13	100							
***SOX*** **-6Bm**	19.27	14.88	14.22	13.72	15.62	11.37	12.35	14.78	15.38	81.43	81.13	100	100						
***SOX*** **-13Bm**	15.66	13.33	12.97	14.50	14.73	13.33	13.72	11.76	14.83	70.58	70.58	65.49	65.49	100					
***SOX*** **-8Bt**	37.34	30.15	33.05	30.19	35.71	32.15	31.32	32.68	39.01	12.50	12.45	12.45	12.45	12.54	100				
***SOX*** **-9BiBt**	39.75	27.86	32.21	29.01	33.48	28.62	27.27	29.57	39.56	11.36	11.36	11.36	11.36	12.54	62.50	100			
***SOX*** **-9Bt**	39.75	27.86	32.21	29.01	33.48	28.62	27.27	29.57	39.56	11.36	11.36	11.36	11.36	12.54	62.50	100	100		
***SOX*** **-10BiBt**	36.74	28.73	33.05	29.80	34.82	30.58	30.26	33.07	39.56	12.64	12.64	12.64	12.64	11.37	59.77	72.03	72.03	100	
***SOX*** **-10Bm**	36.74	28.73	33.05	29.80	34.82	30.58	30.26	33.07	39.56	12.64	12.64	12.64	12.64	11.37	59.77	72.03	72.03	100	100

SRY: Sex-determining region Y, Bt: *Bos taurus*, BiBt: *Bos* indicus x *Bos tautus*, and Bm: *Bos mutus*. Colors were assigned based on the percent pairwise similarity scores of *Sox* sequences with red indicating scores between 11–19; yellow, 30–43 and green, 60–100.

## Data Availability

Data used, and their accession numbers are included in this manuscript. See supplementary Tables S1–S3.

## Supplementary Materials

The following supporting information can be downloaded at: https://www.mdpi.com/article/10.3390/genes13101783/s1, Table S1: Genetic Distance of *Bos Sox* gene subfamilies; Table S2: Amino acid composition of *Sox* genes in *Bos* sp; Table S3: Gene ontology: biological processes, molecular function and cellular components of *Sox* genes in *Bos* sp. (PANTHER); Table S4: Gene ontology: biological processes, molecular function and cellular components of *Sox* genes in *Bos* sp. (DAVID).
