# Supplementary material for: Molecular Evolution and Inheritance Pattern of Sox Gene Family among Bovidae
Source: Genes (Basel). 2022 Oct 2;13(10):1783. doi: 10.3390/genes13101783 (PMC9602320; doi:10.3390/genes13101783)
Supplement: Supplementary file 1 [file genes-13-01783-s001.zip › Supplementary Table S1 Genetic Distance of Bos sp Sox genes subfamilies.pdf]

Supplementary Table S1 Genetic Distance of *Bos* sp *Sox* genes subfamilies

**SRY**	100%																									
**SOX-1Bt**	50%	100%																								
**SOX-2Bt**	51.80%	74.05%	100%																							
**SOX-3BiBt**	51.80%	68.62%	68.20%	100%																						
**SOX-14Bt**	47.59%	46.42%	43.75%	47.76%	100%																					
**SOX-21Bt**	42.77%	45.88%	44.35%	43.92%	71.42%	100%																				
**SOX-4Bt**	36.14%	33.20%	34.30%	32.54%	34.37%	37.25%	100%																			
**SOX-11Bt**	36.74%	31.12%	33.05%	32.54%	34.82%	32.15%	54.47%	100%																		
**SOX-12Bt**	30.72%	38.46%	38.46%	41.75%	34.61%	38.46%	59.89%	56.04%	100%																	
**SOX 5Bt**	16.86%	14.12%	12.13%	13.33%	12.05%	12.54%	12.87%	13.22%	16.48%	100%																
**SOX-5Bm**	16.86%	14.12%	12.13%	13.33%	12.05%	12.54%	12.83%	13.22%	16.48%	100%	100%															
**SOX-6BiBt**	19.27%	14.88%	14.22%	13.72%	15.62%	11.37%	12.35%	14.78%	15.38%	81.43%	81.13%	100%														
**SOX-6Bm**	19.27%	14.88%	14.22%	13.72%	15.62%	11.37%	12.35%	14.78%	15.38%	81.43%	81.13%	100%	100%													
**SOX-13Bm**	15.66%	13.33%	12.97%	14.50%	14.73%	13.33%	13.72%	11.76%	14.83%	70.58%	70.58%	65.49%	65.49%	100%												
**SOX-8Bt**	37.34%	30.15%	33.05%	30.19%	35.71%	32.15%	31.32%	32.68%	39.01%	12.50%	12.45%	12.45%	12.45%	12.54%	100%											
**SOX-9BiBt**	39.75%	27.86%	32.21%	29.01%	33.48%	28.62%	27.27%	29.57%	39.56%	11.36%	11.36%	11.36%	11.36%	12.54%	62.50%	100%										
**SOX-9Bt**	39.75%	27.86%	32.21%	29.01%	33.48%	28.62%	27.27%	29.57%	39.56%	11.36%	11.36%	11.36%	11.36%	12.54%	62.50%	100%	100%									
**SOX-10BiBt**	36.74%	28.73%	33.05%	29.80%	34.82%	30.58%	30.26%	33.07%	39.56%	12.64%	12.64%	12.64%	12.64%	11.37%	59.77%	72.03%	72.03%	100%								
**SOX-10Bm**	36.74%	28.73%	33.05%	29.80%	34.82%	30.58%	30.26%	33.07%	39.56%	12.64%	12.64%	12.64%	12.64%	11.37%	59.77%	72.03%	72.03%	100%	100%							

**SOX-7BiBt**	36.74%	29.38%	34.30%	33.72%	32.14%	27.84%	29.47%	32.68%	37.36%	18.93%	18.86%	19.10%	19.10%	17.64%	29.43%	30.68%	30.68%	30.26%	30.26%	100%						
**SOX-17Bm**	22.28%	27.71%	27.10%	27.10%	22.28%	24.69%	27.71%	30.12%	29.51%	13.25%	13.85%	13.85%	13.85%	12.04%	26.50%	27.10%	27.10%	25.90%	25.90%	46.98%	100%					
**SOX-18BiBt**	35.54%	33.19%	31.38%	34.02%	32.58%	30.29%	31.95%	31.95%	37.36%	16.18%	16.18%	16.59%	16.59%	15.35%	30.70%	32.36%	32.36%	30.29%	30.29%	56.01%	56.62%	100%				
**SOX-15BiBt**	40.36%	59.03%	56.02%	54.21%	49.39%	50.60%	40.36%	41.56%	36.74%	15.06%	15.06%	15.06%	15.06%	13.85%	39.15%	38.55%	38.55%	38.55%	38.55%	37.95%	21.68%	37.95%	100%			
**SOX-15Bm**	40.36%	59.03%	56.02%	54.21%	49.39%	50.60%	40.36%	41.56%	36.74%	15.06%	15.06%	15.06%	15.06%	13.85%	39.15%	38.55%	38.55%	38.55%	38.55%	37.95%	21.68%	37.95%	100%	100%		
**SOX-30BiBt**	18.07%	11.45%	12.97%	13.33%	16.07%	14.90%	11.32%	12.45%	12.08%	23.10%	23.01%	22.64%	22.64%	18.43%	13.20%	14.39%	14.39%	14.55%	14.55%	14.33%	10.24%	13.27%	15.66%	15.66%	100%	
**SOX-30Bm**	18.67%	11.45%	12.97%	13.72%	16.51%	14.90%	11.32%	12.06%	11.53%	22.72%	22.64%	23.01%	23.01%	18.82%	13.20%	14.39%	14.39%	14.17%	14.17%	14.33%	10.24%	13.27%	15.66%	15.66%	99.62%	100%
	**SRY**	**SOX-1Bt**	**SOX-2Bt**	**SOX-3BiBt**	**SOX-14Bt**	**SOX-21Bt**	**SOX-4Bt**	**SOX-11Bt**	**SOX-12Bt**	**SOX5Bt**	**SOX-5Bm**	**SOX-6BiBt**	**SOX-6Bm**	**SOX-13Bm**	**SOX-8Bt**	**SOX-9BiBt**	**SOX-9Bt**	**SOX-10BiBt**	**SOX-10Bm**	**SOX-7BiBt**	**SOX-17Bm**	**SOX-18BiBt**	**SOX-15BiBt**	**SOX-15Bm**	**SOX-30BiBt**	**SOX-30Bm**
