# Supplementary material for: Molecular Evolution and Inheritance Pattern of Sox Gene Family among Bovidae
Source: Genes (Basel). 2022 Oct 2;13(10):1783. doi: 10.3390/genes13101783 (PMC9602320; doi:10.3390/genes13101783)
Supplement: Supplementary file 1 [file genes-13-01783-s001.zip › Supplementary Table S2_Amino acid composition of Sox genes.pdf]

Supplementary Table S2. Amino acid composition of Sox genes.
Ala: Alanine, Cys: Cysteine, Asp: Aspartic Acid, Glu: Glutamic acid, Phe: Phenylalanine, Gly: Glycine, His: Histidine, Ile: Isoleucine, Lys: Lysine, Leu: Leucine, Met: Methionine, Asn: Asparagine, Pro: Proline, Gln: Glutamine, Arg: Arginine, Ser: Serine, Thr: Threonine, Val: Valine, Trp: Tryptophan, Tyr: Tyrosine

	Ala	Cys	Asp	Glu	Phe	Gly	His	Ile	Lys	Leu	Met	Asn	Pro	Gln	Arg	Ser	Thr	Val	Trp	Tyr	Total
SRYBt	6.14	2.63	5.26	5.7	3.51	2.63	3.95	3.07	7.02	7.89	2.19	3.95	5.26	5.7	9.21	10.96	5.26	4.82	1.32	3.51	228
SOX-1Bt	18.97	0.27	2.17	3.52	0.54	15.45	5.96	1.36	4.34	5.96	4.61	2.98	8.4	3.79	4.61	7.59	1.63	3.25	0.81	3.79	369
SOX-2Bm	7.67	1.47	2.95	5.6	0.29	10.32	3.54	1.47	5.31	6.78	5.9	3.54	7.67	4.13	7.67	11.5	4.13	4.13	2.06	3.83	339
SOX-2Bt	8.13	0.31	2.81	3.75	0.63	11.88	3.44	1.25	5.31	6.25	7.81	4.69	8.13	5.63	5.31	11.25	4.38	3.75	0.94	4.38	320
SOX-3BiBt	20.18	0.22	3.55	2.22	0.89	12.64	2.44	1.11	3.77	7.1	3.99	2.22	12.42	3.1	5.1	7.98	3.99	3.1	0.67	3.33	451
SOX-14Bt	10.83	1.25	3.75	3.75	2.5	5.83	3.75	2.5	7.5	8.75	3.75	2.92	11.25	3.33	5.42	9.17	5	2.92	1.25	4.58	240
SOX-21Bt	22.38	0.72	2.53	3.97	2.53	8.66	3.61	1.81	6.86	8.3	3.61	2.17	10.11	1.81	4.33	7.94	1.44	2.53	1.08	3.61	277
SOX-4Bt	14.55	0.83	4.99	4.57	2.49	13.72	2.49	2.08	6.03	6.24	1.66	2.49	6.24	1.25	3.53	19.54	2.08	2.49	0.83	1.87	481
SOX-11Bt	11.75	1.11	7.76	7.1	2.66	11.53	1.55	2	6.87	7.76	2.44	1.55	7.76	3.55	3.99	12.64	2	2.88	0.89	2.22	451
SOX-12Bt	10.83	0.96	6.37	11.46	1.91	12.42	1.59	1.91	4.46	5.73	2.55	0.64	12.74	2.55	9.55	5.1	2.55	3.18	1.91	1.59	314
SOX5Bt	8.52	0.41	4.53	8.24	1.65	6.87	2.2	4.12	7.01	8.24	3.57	3.57	7.83	8.38	4.67	8.79	4.12	4.4	0.41	2.47	728
SOX-5Bm	8.16	0.53	4.14	8.02	1.74	6.95	2.54	3.88	6.95	8.02	3.48	3.74	7.75	8.42	4.68	9.22	4.55	4.41	0.4	2.41	748
SOX-6BiBt	7.5	0.89	4.57	7.37	1.78	5.84	1.78	4.19	6.61	7.75	3.94	3.81	7.37	8.89	5.21	10.04	6.1	3.43	0.64	2.29	787
SOX-6Bm	7.02	0.83	4.52	7.37	1.78	6.54	2.5	4.16	6.66	7.85	3.8	3.8	7.13	8.68	5.47	9.87	5.83	3.33	0.59	2.26	841
SOX-13BiBt	7.48	1.46	4.72	8.13	1.3	5.2	2.76	2.76	6.02	9.76	2.93	3.09	9.92	8.46	5.53	9.43	3.25	5.2	0.49	2.11	615
SOX-13Bm	7.36	1.6	5.12	7.84	1.28	5.28	2.72	2.88	5.92	9.6	2.88	3.04	9.76	8.64	5.6	9.44	3.2	5.28	0.48	2.08	625
SOX-8Bt	12.17	1.12	5.24	4.31	1.69	10.86	4.87	1.12	3.75	6.37	1.69	1.31	10.3	3.93	5.99	11.61	5.06	3.75	1.12	3.75	534
SOX-8Bm	8.47	2.39	3.78	5.18	1	10.96	4.08	1.59	4.08	8.67	0.6	1.79	12.25	5.68	9.26	7.87	5.48	3.39	1.49	1.99	1004
SOX-9BiBt	8.02	0.38	4.2	5.92	2.1	6.49	4.01	2.1	4.77	4.77	2.29	2.67	16.98	8.21	4.01	9.35	4.96	4.2	0.95	3.63	524
SOX-9Bt	8.02	0.38	4.2	5.92	2.1	6.49	4.01	2.1	4.77	4.77	2.29	2.67	16.98	8.21	4.01	9.35	4.96	4.2	0.95	3.63	524
SOX-10BiBt	7.89	0.64	5.12	6.18	1.71	11.94	4.69	2.13	5.12	6.18	1.92	1.92	11.3	5.12	4.26	10.66	3.84	4.48	1.07	3.84	469
SOX-10Bm	8.32	0.44	5.03	5.03	1.75	11.6	4.81	2.41	5.47	5.91	1.97	1.97	11.38	5.69	4.6	9.85	4.6	4.16	1.09	3.94	457
SOX-7BiBt	8.53	1.55	4.91	5.94	1.81	9.3	3.36	0.78	3.62	10.59	2.33	2.58	12.4	4.13	5.94	10.34	3.36	3.36	0.78	4.39	387
SOX-7Bm	8.84	1.61	4.82	5.22	1.2	8.43	3.21	0.4	4.42	10.84	2.41	2.41	10.44	5.22	5.62	10.84	4.02	4.02	0.4	5.62	249
SOX-17BiBt	13.17	1.46	4.63	6.1	2.44	11.95	3.17	0.24	3.41	9.02	3.17	1.22	16.34	3.41	5.37	5.37	1.22	3.9	0.73	3.66	410
SOX-17Bm	11.57	1.85	3.7	7.41	2.78	10.65	2.78	0.93	3.7	8.33	1.85	3.24	12.04	1.85	9.26	7.41	2.31	4.63	1.39	2.31	216
SOX-18BiBt	14.65	1.8	4.11	6.68	2.57	8.23	1.03	0.77	2.83	9.51	1.29	1.54	19.28	2.06	8.74	5.91	2.06	2.83	1.03	3.08	389
SOX-15BiBt	7.3	0.86	2.15	4.29	1.72	11.16	2.58	0.43	4.72	7.73	2.15	3.43	14.59	5.15	6.87	12.02	3	3.43	1.72	4.72	233
SOX-15Bm	7.3	0.86	2.15	4.29	1.72	11.16	2.58	0.43	4.72	7.73	2.15	3.43	14.59	5.15	6.87	11.59	3.43	3.43	1.72	4.72	233
SOX-30BiBt	9.53	1.36	1.75	7.59	1.95	5.45	1.75	4.28	5.64	9.92	1.36	2.72	14.4	5.45	7.39	6.61	5.06	5.84	0.58	1.36	514
SOX-30Bm	8.13	1.26	2.66	7.15	2.81	5.19	3.09	3.93	4.35	8.42	0.98	3.09	13.88	4.91	6.17	9.82	5.47	5.33	0.42	2.95	713
Avg	9.86	1.07	4.21	6.26	1.78	8.72	3.08	2.39	5.32	7.79	2.73	2.74	10.97	5.76	5.82	9.6	4.1	3.95	0.89	2.97	473.2
