# Supplementary material for: Molecular Evolution and Inheritance Pattern of Sox Gene Family among Bovidae
Source: Genes (Basel). 2022 Oct 2;13(10):1783. doi: 10.3390/genes13101783 (PMC9602320; doi:10.3390/genes13101783)
Supplement: Supplementary file 1 [file genes-13-01783-s001.zip › Supplementary Table S3 Gene Ontology.pdf]

Supplementary Table S3**.** Gene Ontology biological processes, molecular functions, and cellular components of *Bos Sox* genes

| **GO biological process complete** | Gene | Pvalue | FDR |
|---|---|---|---|
| metanephric nephron tubule formation (GO:0072289) | 2 | 3.22E-06 | 5.59E-04 |
| renal vesicle induction (GO:0072034) | 2 | 3.22E-06 | 5.52E-04 |
| positive regulation of mesenchymal stem cell differentiation (GO:2000741) | 2 | 5.37E-06 | 8.39E-04 |
| endocardium formation (GO:0060214) | 2 | 5.37E-06 | 8.30E-04 |
| metanephric tubule formation (GO:0072174) | 2 | 5.37E-06 | 8.22E-04 |
| stem cell fate specification (GO:0048866) | 2 | 5.37E-06 | 8.13E-04 |
| astrocyte fate commitment (GO:0060018) | 2 | 8.05E-06 | 1.16E-03 |
| endocardial cell differentiation (GO:0060956) | 2 | 8.05E-06 | 1.15E-03 |
| cardiac endothelial cell differentiation (GO:0003348) | 2 | 8.05E-06 | 1.14E-03 |
| endocardium morphogenesis (GO:0003160) | 2 | 8.05E-06 | 1.12E-03 |
| metanephric nephron tubule morphogenesis (GO:0072282) | 2 | 1.13E-05 | 1.49E-03 |
| lacrimal gland development (GO:0032808) | 2 | 1.13E-05 | 1.47E-03 |
| common bile duct development (GO:0061009) | 2 | 1.13E-05 | 1.46E-03 |
| metanephric tubule morphogenesis (GO:0072173) | 2 | 1.50E-05 | 1.86E-03 |
| stem cell fate commitment (GO:0048865) | 2 | 1.50E-05 | 1.85E-03 |
| positive regulation of kidney development (GO:0090184) | 2 | 1.50E-05 | 1.83E-03 |
| negative regulation of photoreceptor cell differentiation (GO:0046533) | 2 | 1.50E-05 | 1.81E-03 |
| regulation of photoreceptor cell differentiation (GO:0046532) | 2 | 1.50E-05 | 1.80E-03 |
| regulation of mesenchymal stem cell differentiation (GO:2000739) | 2 | 1.93E-05 | 2.27E-03 |
| ureter morphogenesis (GO:0072197) | 2 | 1.93E-05 | 2.26E-03 |
| Sertoli cell development (GO:0060009) | 2 | 2.41E-05 | 2.69E-03 |
| endocardium development (GO:0003157) | 2 | 2.41E-05 | 2.67E-03 |
| limb bud formation (GO:0060174) | 2 | 2.94E-05 | 3.11E-03 |
| glial cell fate commitment (GO:0021781) | 2 | 3.53E-05 | 3.63E-03 |
| ureter development (GO:0072189) | 3 | 2.92E-07 | 6.57E-05 |
| metanephric nephron tubule development (GO:0072234) | 2 | 4.17E-05 | 4.20E-03 |
| positive regulation of chondrocyte differentiation (GO:0032332) | 3 | 4.08E-07 | 8.75E-05 |
| metanephric tubule development (GO:0072170) | 2 | 4.87E-05 | 4.83E-03 |
| sex determination (GO:0007530) | 3 | 4.75E-07 | 1.01E-04 |
| metanephric nephron epithelium development (GO:0072243) | 2 | 5.61E-05 | 5.53E-03 |
| retinal rod cell differentiation (GO:0060221) | 2 | 5.61E-05 | 5.49E-03 |
| positive regulation of branching involved in ureteric bud morphogenesis (GO:0090190) | 2 | 5.61E-05 | 5.45E-03 |
| enteric nervous system development (GO:0048484) | 2 | 5.61E-05 | 5.42E-03 |
| male sex determination (GO:0030238) | 2 | 5.61E-05 | 5.38E-03 |
| Sertoli cell differentiation (GO:0060008) | 2 | 6.41E-05 | 6.11E-03 |
| regulation of branching involved in ureteric bud morphogenesis (GO:0090189) | 2 | 7.26E-05 | 6.83E-03 |
| positive regulation of stem cell differentiation (GO:2000738) | 2 | 8.17E-05 | 7.58E-03 |
| metanephric nephron morphogenesis (GO:0072273) | 2 | 9.12E-05 | 8.41E-03 |
| metanephric epithelium development (GO:0072207) | 2 | 9.12E-05 | 8.36E-03 |

| | | | |
|---|---|---|---|
| nephron tubule formation (GO:0072079) | 2 | 1.01E-04 | 9.23E-03 |
| positive regulation of cartilage development (GO:0061036) | 3 | 1.45E-06 | 2.85E-04 |
| negative regulation of myoblast differentiation (GO:0045662) | 2 | 1.35E-04 | 1.20E-02 |
| regulation of stem cell proliferation (GO:0072091) | 3 | 2.53E-06 | 4.45E-04 |
| regulation of kidney development (GO:0090183) | 2 | 1.47E-04 | 1.30E-02 |
| metanephros morphogenesis (GO:0003338) | 2 | 1.60E-04 | 1.38E-02 |
| oligodendrocyte differentiation (GO:0048709) | 4 | 7.00E-08 | 1.74E-05 |
| metanephric nephron development (GO:0072210) | 2 | 2.01E-04 | 1.64E-02 |
| positive regulation of morphogenesis of an epithelium (GO:1905332) | 2 | 2.01E-04 | 1.63E-02 |
| developmental induction (GO:0031128) | 2 | 2.01E-04 | 1.62E-02 |
| **GO molecular function complete** | | | |
| beta-catenin binding (GO:0008013) | 3 | 2.65E-05 | 6.61E-03 |
| DNA-binding transcription activator activity, RNA polymerase II-specific (GO:0001228) | 8 | 3.12E-10 | 8.76E-08 |
| DNA-binding transcription activator activity (GO:0001216) | 8 | 3.36E-10 | 8.89E-08 |
| RNA polymerase II cis-regulatory region sequence-specific DNA binding (GO:0000978) | 18 | 9.28E-25 | 4.17E-21 |
| cis-regulatory region sequence-specific DNA binding (GO:0000987) | 18 | 1.32E-24 | 2.98E-21 |
| DNA-binding transcription factor activity, RNA polymerase II-specific (GO:0000981) | 18 | 6.41E-24 | 9.61E-21 |
| RNA polymerase II transcription regulatory region sequence-specific DNA binding (GO:0000977) | 18 | 1.06E-23 | 1.20E-20 |
| DNA-binding transcription factor activity (GO:0003700) | 18 | 1.72E-23 | 1.54E-20 |
| transcription cis-regulatory region binding (GO:0000976) | 18 | 3.77E-23 | 2.83E-20 |
| transcription regulatory region nucleic acid binding (GO:0001067) | 18 | 3.82E-23 | 2.46E-20 |
| sequence-specific double-stranded DNA binding (GO:1990837) | 18 | 7.82E-23 | 4.40E-20 |
| double-stranded DNA binding (GO:0003690) | 18 | 2.44E-22 | 1.22E-19 |
| sequence-specific DNA binding (GO:0043565) | 18 | 2.78E-22 | 1.25E-19 |
| transcription regulator activity (GO:0140110) | 18 | 3.37E-21 | 1.38E-18 |
| DNA binding (GO:0003677) | 18 | 5.82E-19 | 2.18E-16 |
| nucleic acid binding (GO:0003676) | 18 | 2.25E-15 | 7.79E-13 |
| heterocyclic compound binding (GO:1901363) | 18 | 1.08E-11 | 3.48E-09 |
| organic cyclic compound binding (GO:0097159) | 18 | 1.38E-11 | 4.15E-09 |
| binding (GO:0005488) | 18 | 3.38E-05 | 8.00E-03 |
| **GO cellular component complete** | | | |
| transcription regulator complex (GO:0005667) | 6 | 7.39E-07 | 6.94E-04 |
| nucleus (GO:0005634) | 18 | 2.33E-10 | 4.38E-07 |
| intracellular membrane-bounded organelle (GO:0043231) | 18 | 1.57E-06 | 9.86E-04 |
| membrane-bounded organelle (GO:0043227) | 18 | 3.34E-06 | 1.57E-03 |
| intracellular organelle (GO:0043229) | 18 | 1.51E-05 | 5.69E-03 |
| organelle (GO:0043226) | 18 | 3.48E-05 | 1.09E-02 |
