# Supplementary material for: Molecular Evolution and Inheritance Pattern of Sox Gene Family among Bovidae
Source: Genes (Basel). 2022 Oct 2;13(10):1783. doi: 10.3390/genes13101783 (PMC9602320; doi:10.3390/genes13101783)
Supplement: Supplementary file 1 [file genes-13-01783-s001.zip › Supplementary Table S4 Gene Ontology DAVID.pdf]

Supplementary Table S4: Gene ontology biological process, cellular component and molecular function of Sox genes (DAVID)

	Genes	Pvalue
**Cellular component**		
Nuclear transcription factor, complex	SOX2, 1, 14, 21	6.20E-12
nucleoplasm	SOX2, 6, 10, 4	4.60E-02
**Molecular function**		
Transcription regulatory region DNA bonding	SOX2, 17, 6, 7, 30	1.40E-04
sequence specific DNA binding	SOX18, 8, 10	2.10E-04
transcription factor activity, RNA	SOX17, 7, 10	7.10E-04
transcription factor activity, sequence specific	SOX2, 6, 21, 4	7.40E-03
transcriptional activator activity , RNA	SOX1, 18, 4	1.20E-02
RNA polymerase II core promoter	SOX1, 18, 10	2.50E-02
core promoter sequence-specific DNA binding	SOX1, 4	3.00E-02
**Biological process**		
cell maturation	SOX18, 8, 6, 10	4.60E-04
in utero embryonic development	SOX18, 8, 6, 10	5.00E-04
positive regulation of gliogenesis	SOX8, 10	2.00E-03
stem cell fate specification	SOX17, 18	2.00E-03
positive regulation of transcription from	SOX2, 15, 8, 6, 4	2.20E-03
positive regulation of mesenchyma stem	SOX6,5	3.00E-03
morphogenesis of branching epithelium	SOX8,10	3.00E-03
negative regulation of canonical	SOX2, 17, 10	3.40E-03
endocardium formation	SOX17, 18	4.00E-03
endocardial cell differentiation	SOX17, 18	5.10E-03
regulation of stem cell proliferation	SOX17, 18	6.10E-03
negative regulation of transcription from	SOX17, 18, 15, 6	7.90E-03
mRNA transcription from RNA polymerase	SOX17, 18	1.00E-02
enteric nervous system development	SOX8, 10	1.10E-02
peripheral nervous system development	SOX8, 10	1.10E-02
positive regulation of chondrocyte differentiation	SOX6,5	1.20E-02
spermatogenesis	SRY, SOX17, 8, 30	1.50E-02
oligodendrocyte differentiation	SOX8, 10	1.70E-02
somatic stem cell population maintenance	SOX2, 4	2.60E-02
cellular response to transforming growth factor	SOX6,5	2.80E-02
hair follicle development	SOX18, 21	3.10E-02
neural crest cell migration	SOX8, 10	4.40E-02
vasculogenesis	SOX17, 18	4.80E-02
canonical Wnt signaling pathway	SOX17, 4	6.70E-02
osteoblast differentiation	SOX2, 8	8.60E-02
protein stabilization	SOX17, 4	9.90E-02
